# Topical Nano Clove/Thyme Gel against Genetically Identified Clinical Skin Isolates: In Vivo Targeting Behavioral Alteration and IGF-1/pFOXO-1/PPAR γ Cues

**DOI:** 10.3390/molecules26185608

**Published:** 2021-09-15

**Authors:** Jilan A. Nazeam, Ghada M. Ragab, Amira A. El-Gazar, Shereen S. El-Mancy, Lina Jamil, Sahar M. Fayez

**Affiliations:** 1 Pharmacognosy Department, Faculty of Pharmacy, October 6 University, Giza 12585, Egypt; 2 Pharmacology and Toxicological Department, Faculty of Pharmacy, Misr University, Giza 12585, Egypt; ghadaragab1311@gmail.com; 3 Pharmacology and Toxicological Department, Faculty of Pharmacy, October 6 University, Giza 12585, Egypt; dr.amira.ams@gmail.com; 4 Pharmaceutics Department, Faculty of Pharmacy, October 6 University, Giza 12585, Egypt; shereenelmancy@o6u.edu.eg (S.S.E.-M.); saharmfayez@o6u.edu.eg (S.M.F.); 5 Microbiology and Immunology Department, Faculty of Pharmacy, October 6 University, Giza 12585, Egypt; lina.jamil@gmail.com

**Keywords:** antibiotic, clinical bacterial isolate, clove, IGF-1, nanogel, non-antibiotic, PPAR γ, thyme, skin infection, essential oil, *Pseudomonas stutzeri*, *Enterococcus faecium*, *Bacillus thuringiensis*

## Abstract

Antimicrobial resistance is a dramatic global threat; however, the slow progress of new antibiotic development has impeded the identification of viable alternative strategies. Natural antioxidant-based antibacterial approaches may provide potent therapeutic abilities to effectively block resistance microbes’ pathways. While essential oils (EOs) have been reported as antimicrobial agents, its application is still limited ascribed to its low solubility and stability characters; additionally, the related biomolecular mechanisms are not fully understood. Hence, the study aimed to develop a nano-gel natural preparation with multiple molecular mechanisms that could combat bacterial resistance in an *acne vulgaris* model. A nano-emulgel of thyme/clove EOs (NEG8) was designed, standardized, and its antimicrobial activity was screened in vitro and in vivo against genetically identified skin bacterial clinical isolates (*Pseudomonas stutzeri*, *Enterococcus faecium* and *Bacillus thuringiensis*). As per our findings, NEG8 exhibited bacteriostatic and potent biofilm inhibition activities. An in vivo model was also established using the commercially available therapeutic, adapalene in contra genetically identified microorganism. Improvement in rat behavior was reported for the first time and NEG8 abated the dermal contents/protein expression of IGF-1, TGF-β/collagen, Wnt/β-catenin, JAK2/STAT-3, NE, 5-HT, and the inflammatory markers; p(Ser536) NF-κBp65, TLR-2, and IL-6. Moreover, the level of dopamine, protective anti-inflammatory cytokine, IL-10 and PPAR-γ protein were enhanced, also the skin histological structures were improved. Thus, NEG8 could be a future potential topical clinical alternate to synthetic agents, with dual merit mechanism as bacteriostatic antibiotic action and non-antibiotic microbial pathway inhibitor.

## 1. Introduction

Antimicrobial resistance (AMR) is one of the top ten global health threats facing humanity, requiring urgent multi-sectorial action to achieve sustainable development goals [1]. The invention of new natural antibiotics and therapies has long been advocated by dermatologists and microbiologists as a strategy to overcome the slow-growing production of synthetic agents [2]. Moreover, the use of non-antibiotic agents is a new approach that warranted to combat microbial resistance by altering the pathogenesis or physiological effects of microbial species [3].

Since the 1990s, misaligned economic incentives have slowed the development of novel antibiotics as fewer pharmaceutical companies have engaged in the process of drug development [4]. In clinical settings, approximately 90% of antibiotics are derived from natural sources, which are composed of a privileged group of structures that interact with a wide range of protein targets [5]. Natural antioxidants could play a vital role as antibiotics, where they not only affect bacteria but also boost human immunity for a long-lasting period [6]. According to Dreger et al., 2014, there are about 3000 essential oils (EOs) that are currently identified, with only 10% having commercial importance [7]; therefore, new application areas are yet to be discovered. Despite the reported potential of EOs as alternatives to synthetic antibiotics, which pose higher risks to human health, there is an industrial limited application of EOs. These hindrances are attributed to low water solubility, stability, volatility, and sensory perception, which become altered due to heat, oxidation, or chemical interactions. Nevertheless, the nanotechnology approach may represent viable approach to overcome these challenges [8], where it is considered as a promising strategy to enhance solubility and skin penetration, and increase long-term safety and efficacy [9].

Skin infection is a common dermal disease that is widely spread in communities and hospitals. Infection severity can range from the superficial to intense deep tissue attack, potentially increasing morbidity in untreated patients [10]. Additionally, many factors such as environment and lifestyle can negatively affect skin health, which, in turn, could result in the spread of communicable dermal disorders such as acne, furuncles, carbuncles, and impetigo [11]. Acne vulgaris is estimated to affect 9.4% of the global population, making it the eighth most prevalent global disease worldwide [12]. Many quantitative and qualitative studies have demonstrated the impact of acne and topical treatments on health-related quality life measures [13]. Crucially, quantitative information about the specific acne antibiotics treatments is very limited, although the dermatology community has an enormous antibiotic load [14]. Routinely prolonged single local antibiotic courses for 3–6 months may potentiate resistance. Previous reports indicated that more than 50% of *P. acnes* were found to be resistant to topical macrolides, making them less effective [15]. Hence, in this study, we used the acne skin disorder as a model of skin bacterial resistance.

The research hypothesis supports the use of EOs as a natural alternative business model that can force sustainable manufacturing the norm for the topical antibiotics industry [16,17]. The study aimed to develop a new antimicrobial agent from essential oils by using nanotechnology to enhance EOs’ physicochemical characters and potentiate its efficacy. Moreover, the underlying molecular pathways were dissected to investigate the non-antibiotic modes of action against resistant skin infections diseases like acne. In this regard, in vivo antioxidant, anti-inflammatory, genetic mechanism, animal behavior, and histological indices were explored. Molecularly, tracking of IGF-1/pFOXO-1/PPAR γ, TGF-β/collagen production, canonical Wnt/β- catenin, JAK-2/STAT-3 cytokine signaling, dopamine, serotonin, norepinephrine, and collagen type 1 for the designed bioactive nano-emulgel were estimated.

## 2. Material and Methods

### 2.1. Essential Oil (EOs) Study Samples

Cinnamon (*Cinnamomum zelylanicum),* cumin (*Cuminum cyminum*), clove (*Syzygium aromaticum*), eucalyptus (Eucalyptus globulus), garlic (*Allium sativum*), thyme (*Thymus vulgaris*), peppermint (*Mentha piperita*), lavender (*Lavendula anguestifolia*), geranium (Pelargonium graveolens), and lemongrass (*Cymbopogon citratus*) were obtained from National Research Center, Dokki, Cairo. The oils were prepared by steam distillation and used as a pharmaceutical-grade essential oils sample. Performance standards for antimicrobial susceptibility testing was according to clinical and laboratory standards institute (CLSI) document M100-S20 (ISBN 1-56238-716-2), 940 West Valley Road, Suite 1400, Wayne, Pennsylvania 19087-1898 USA, 2010: twentieth informational supplement.

### 2.2. Authentication of Commercial EOs

Essential oil samples were authenticated and standardized by gas chromatography-mass spectrometry (GC-MS) using TRACE GC Ultra Gas Chromatographs (THERMO Scientific Corp., Waltham, MA, USA), coupled with a THERMO mass spectrometer detector (ISQ Single Quadrupole Mass Spectrometer). The relative percentages of EOs constituents were evaluated from the total peak areas and identified by comparing of their mass spectra with authentic chemicals (the NSIT library, Wiley spectral library collection).

### 2.3. Antioxidant Determination Using 2,2-Diphenyl-1-Picrylhydrazyl (DPPH) and Ferric Reducing Antioxidant Power (FRAP) Analyses

The antioxidant activity of the EOs was measured using the 1,1-diphenyl-2-picrylhydrazyl (DPPH) free radical scavenging assay and compared with that of ascorbic acid (vitamin C) [18]. The FRAP test was conducted according to Olszowy et al. [19].

### 2.4. Development of Nano-Emulgels (NEG)

Nine nano-emulsion systems (NEG) were prepared by mixing variable contents of isopropyl myristate (IPM) as oily phase IPM, water, and a fixed surfactant mixture concentration (60% *w*/*w*) of Tween 80 and Cremophor RH-40 in different ratios (1:1, 2:1, and 3:1) as shown in (Table 1). The systems were loaded with a mixture of the most bioactive antioxidant oils (30 mg/mL) and consequently evaluated by visual inspection for optical clarity, homogeneity, phase separation, and consistency. The system showed clear nano-emulsion gels (NEGs), which were then subjected to further characterization.

#### Characterization of NEGs and Selected Formula

Particle size (PS), polydispersity index (PDI), and zeta potential (ZP) of NEGs were assessed by zetasizer (ZS, Malvern Instruments Ltd., Malvern, UK) after proper NEG dilution, the measurements were performed in triplicates at room temperature (25 ± 2 °C). A spread ability test was also conducted by pressing 0.5 g of each NEG formula between two glass slides and waiting 5 min until no more spreading was expected, then, the formed circles were measured. Next, the pH values of gels were measured using a calibrated pH meter. The formula showing the most optimized characteristics was morphological analyis using a transmission electron microscope (TEM). The selected NEG was investigated for its intrinsic stability and stored in a sealed clean glass vial at ambient conditions for 6 months. It was visually inspected and analyzed for PS, PDI, and ZP [20].

### 2.5. Antibacterial Bioassays

#### 2.5.1. Bacterial Culturing and Identification

The antibacterial activity of the selected NEG8 with thymol and clove EOs that possessing the highest antioxidant activity was screened against clinically isolated bacteria [21]. The gram stain test was used to identify the following three isolated bacteria from a skin infection clinical situation; B1, Gram-negative rods; B2, Gram-positive cocci; and B3, Gram-positive bacilli in pure cultures according to Gehardt et al. [22].

#### 2.5.2. Biochemical Identification of Bacterial Isolates Using Vitek2 Compact System

Identification by Vitek2 compact system was established according to the manufacture’s instruction (Biomeriux VITEK-2 Compact ref Manual—Ref-414532). A transmittance optical system allows interpretation of test reactions using different wavelengths in the visible spectrum. During incubation, each test reaction was read every 15 min to measure either turbidity or coloured products of substrate metabolism. In addition, a special algorithm was used to eliminate false reading due to small bubbles that may present.

#### 2.5.3. Molecular Identification of Clinically Isolated Bacteria

##### DNA Extraction

Samples of DNA extraction of B1 and B2 were performed using the GeneJet DNA extraction kit (Thermo Fisher Scientific, 5791 Van Allen Way, Carlsbad, CA, USA). Nucleic acid was later eluted in 100 µL of elution buffer [23].

##### Analysis of Polymerase Chain Reaction (PCR) Products

PCR products were separated by using electrophoresis on 1.5% agarose gel (Applichem, GmbH, Darmstadt, Germany) electrophoresis. Fragment sizes were calibrated using a GelPilot 100 base pair (bp) plus ladder). Gels were photographed using a gel documentation system (Alpha Innotech, Biometra, Germany) and the data were analyzed through computer software.

##### Microbial Sequencing

PCR products were purified using the QIAquick PCR Product extraction kit (Qiagen, Valencia, Spain). A Bigdye Terminator V3.1 cycle sequencing kit (Perkinelmer, Foster City, CA, USA) was used to perform sequencing reactions, which was purified using Centrisep spin column. DNA sequences were obtained by Applied Biosystems 3130 genetic analyzer (HITACHI, Tokyo, Japan). BLAST® (Basic Local Alignment Search Tool) analysis [24] was initially performed to establish sequence identity to GenBank accessions [25]. The database alignment using NCBI-BLAST was analyzed computationally to generate a phylogenetic tree. Oligonucleotide primers for bacteria were supplied from Metabion (Planegg, Germany) [Supplementary Materials].

#### 2.5.4. Bioactivity Assay Using the Cup Agar Diffusion Method

A sensitivity test was established using the cup agar diffusion method [26]. Bacterial cultures were diluted at 1:100 (≈10^6^ colony forming units (CFU)/mL), the brain heart infusion agar (BHA) plates were inoculated with 100 µL of each bacterial isolate and 5 mm pores filled with 100 mL of the selected NEG8 at 10 mg/mL. The plates were incubated for 24 h at 37 °C; thereafter, inhibition zones were measured (mm).

#### 2.5.5. Determination of Minimum Inhibitory Concentration (MIC) and Minimum Bactericidal Concentration (MBC)

The MIC of NEG8 was evaluated using serial dilution method according to CLSI. A series of tubes was prepared with a broth medium to which various concentrations of the NEG8 were added. The tubes were then inoculated with 0.5 mL of McFarland standard of the isolated bacterial suspensions. Bacterial suspensions were used as negative control, while broth containing NEG8 was used as positive control. After incubation at 35 ± 2 °C, the tests were examined, and the MIC was determined. Then, aliquots of 0.5 μL from all the tubes which showed no visible bacterial growth were seeded on BHA agar plates and incubated for 24 h at 37 °C. The MBC was defined as the lowest concentration of NEG8 that was able to prevent microbial growth in a culture medium [27].

#### 2.5.6. Biofilm Inhibition Assay, Quantification, and Percentage of Inhibition

The biofilms were assayed, and evaluated according to Stepanovic et al., 2007 [28]. The ability of the optimized NEG8 to inhibit the biofilm of isolated bacteria (B1, B2, B3) was evaluated as modified method of O’Toole, G.A [29]. The quantitative analysis of biofilm production was performed by adding 125 µL of 30% acetic acid to de-stain the samples. Afterward, the optical density (OD) at 620 nm was assessed using microplate ELIZA reader (FLUOstar Omega, BMG, Labtech, Ortenberg, Germany). The percentage of biofilm inhibition was determined by the formula:

$$\%\mathrm{Inhibition}\;=\frac{\mathrm{OD}\;\mathrm{control}\;-\;\mathrm{OD}\;\mathrm{sample}}{\mathrm{OD}\;\mathrm{control}}\times 100$$

### 2.6. In Vivo Anti-Skin Infection Potential of the Optimized Selected NEG

Animal studies were approved and followed the guidelines of the laboratory animal ethics guide for the care and use of laboratory animals agreed by the National Institutes of Health (NIH publication, 1996). The in vivo antibacterial activity of the most optimized nanogel NEG was evaluated using albino rats. Injection sites were shaved, and the left ear was pre-fixed to shelf using double-sided tape. This was followed by an intradermal (i.d.) injection of 10 μL of bacterial cultures (B1, B2, B3) to the dorsal site of the ear. Similarly, 10 μL of phosphate buffered saline (PBS) was injected into the right ear of the same rat and considered as a negative control group [30]. Then, 24 h later, the optimized nanogel preparation (NEG8) and commercially available standard adapalene gel (ADA) (250 mg/kg) [31] were topically applied to their animal groups once daily for four consecutive days; its antibacterial potential was then evaluated.

Experimental design: the rats were divided into the following 10 groups (*n* = 10). (1) G.I, negative control group; (2) G.II, treated with B1; (3) G.IIa, treated with B1 + NEG8; (4) G.IIb, treated with B1 + ADA; (5) G.III, treated with B2; (6) G.IIIa, treated with B2 + NEG8; (7) G.IIIb, treated with B2 +ADA; (8) G.IV, treated with (B3); (9) G.Iva, treated with B3 + NEG8; and (10) G.IVb, treated with B3 + ADA.

#### 2.6.1. Behavioral Open Field Tests

The tests commenced 24 h after the end of the experiment. Ten rats (first set) were subjected to the open field test, which measured latency time (time spent from placing the animal into the middle of the device until its first movement), ambulation frequency (number of squares crossed by the animal over 3 min), and rearing frequency (number of times the animal stood extended and extended on rear appendages with no forelimb support) [32].

#### 2.6.2. Western Blotting

Western blotting technique was performed as previously described [33]. The optical density (OD) of the subsequently mentioned results was normalized to β-actin and total protein.

#### 2.6.3. ELISA Parameters

ELISA kits were used to measure homogenate levels of insulin-like growth factor-1 (IGF-I), p(Ser256) Forkhead box protein O-1 (p(Ser256)FOXO-1), IL-6, IL-10, dopamine, serotonin, norepinephrine, collagen type 1, and transforming growth factor β-1 (TGFβ-1) levels according to the manufacturer’s instructions.

#### 2.6.4. RT-PCR Analysis

Tissue specimens were generated as previously described by Je et al., 2020 [33]. Quantitative converse transcriptase (qRT) lists are provided in the supplementary materials. The expression levels were analyzed by Real-Time StatMiner (Integromics, Madrid, Spain). β-actin levels were used as an internal control, and fold changes were calculated by relative quantification.

#### 2.6.5. Histopathological Examinations

Skin samples were collected from three rats per group, that were embedded in paraffin wax for sectioning and staining with hematoxylin and eosin (H&E) using the Leica application module for tissue section analysis (Leica Microsystems GmbH, Wetzlar, Germany).

### 2.7. Statistical Analysis

Data were analyzed using analysis of variance (ANOVA), followed by Tukey’s post hoc test. All the values were expressed as the mean ± S.D. The inhibition of bacterial biofilms at different concentrations of optimized NEG was compared by two-way ANOVA (Bonferroni post hoc tests). GraphPad Prism software (version 5.0d; GraphPad Software, Inc., San Diego, CA, USA) was used for all statistical analyses, *p* < 0.05 value was considered statistically significant.

## 3. Results and Discussion

### 3.1. GC-MS Chromatographic Analyses

The authentication and standardization of EO samples was established by comparing the components retention time and mass spectra with published data (Table 2). Quantitative analysis of thyme and clove oil showed that thymol (65.6%) and eugenol (94.3%) was the major principle of thyme and clove oil, respectively.

### 3.2. Antioxidant Activity of EOs

Thanks to increased knowledge, consumers are gradually moving toward natural antioxidants with no synthetic ingredients. In addition to the cosmetic and preservative activities of EOs, its lipophilic, small non-polar molecules are considered as natural skin penetration enhancers, which support the increasing interest in natural oil research [34]. The antioxidant activity of tested essential oils was evaluated using DPPH free radical scavenging assays and FRAP.

**Figure 1:**
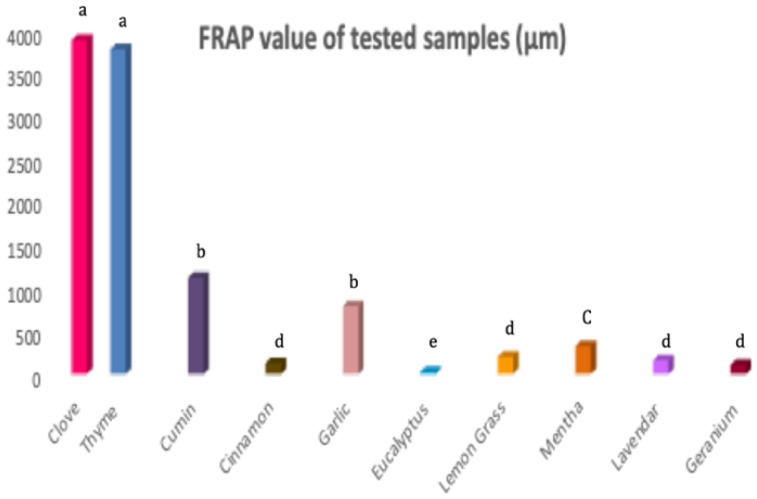
FRAP assay of different essential oils samples. Letters indicate statistical differences in antioxidant activity between different oils (Tukey test, *p* < 0.05).

**Figure 2:**
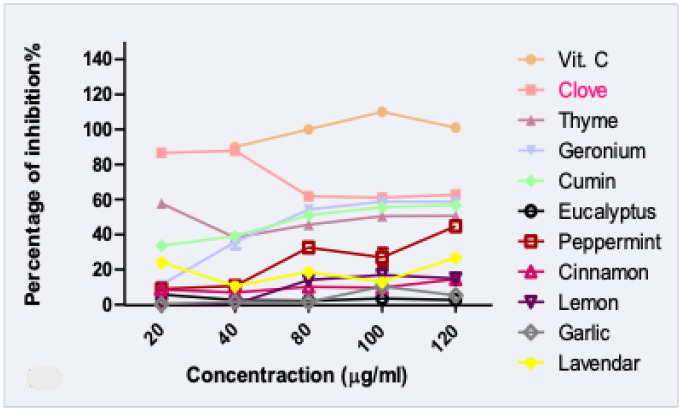
DPPH assay of different essential oil samples.

The results (Figure 1 and Figure 2) showed that thyme and clove oils possessed the highest antioxidant activities [35,36]. Clove and vitamin C exerted the same percentage of antioxidant activity and were insignificantly different at a concentration of 40 µg/mL. However, all the tested oils showed a significant difference to ascorbic acid at concentration 80, 100, 120 µg/mL (*p* < 0.05). Moreover, the strongest activity reducing ion Fe^3+^ to Fe^2+^ was noted for thyme and clove oil. Both most bio-active EOs were previously characterized by a high percentage of phenols compounds, that potentially contributed to dual free radical scavenging and antimicrobial activities [37]. Hence, the clove and thyme oils were used for preparing functional nano-emulgel (NEGs), to improve oils physicochemical characters and investigate the synergistic antimicrobial activity against acne clinical bacterial isolates.

### 3.3. Characterization of the Prepared NEGs

**Figure 3:**
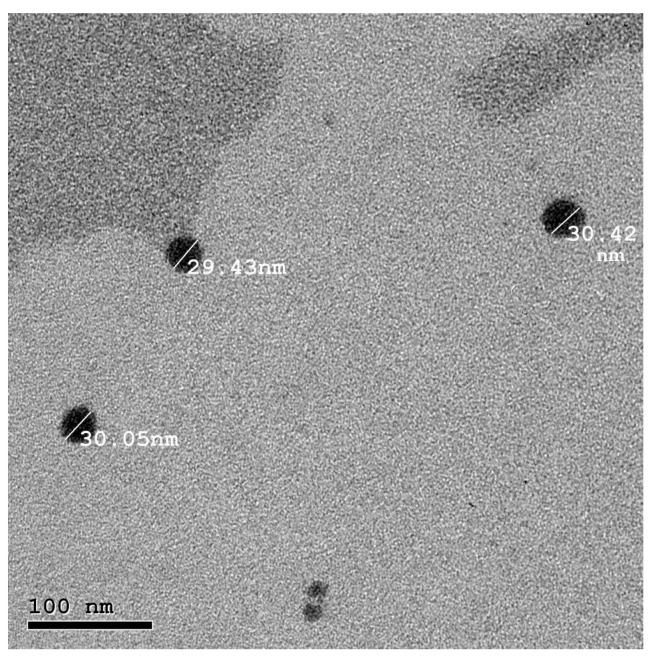
Transmission electron micrograph of the selected NEG8.

Visual inspection revealed that formulae NEG1, NEG4, and NEG7 (highest water and the lowest oil content) were turbid, while NEG3, NEG6, and NEG9 (lowest water and the highest oil content) were transparent yellow fluids, so they were excluded. Formulae NEG2, NEG5, and NEG8, which were prepared using equal amounts of oil and water, were noted to have displayed a proper yellow transparent gel-like consistency; hence, these formulae were selected for further characterization. The pH values of the selected formulae ranged from 5 to 5.96, which are compatible with the pH of the skin and suitable for topical application. A spreadability test, which is vital for patient compliance, was performed. Good spreadability aids in easy and uniform application of topical skin preparation [38]. The spreadability values for the selected NEGs varied between 6.89 ± 0.12 and 7.4 ± 0.26 cm, indicating convenient spreading properties. PS analyses revealed that NEG8 had the smallest size (33.46 ± 1.315 nm) (Table 3). In term of PDI, the ratio of SD to mean PS, was 0.315 ± 0.05. A low PDI value indicates good uniformity of the PS in the formula [39]. ZP data for NEG8 reflects good stability (−14.2 ± 2.316 mV), and TEM confirmed that NEG8 particles were nanometer in size (30 nm), of uniform spherical shape with no aggregation (Figure 3). Based on these data, NEG8 was selected for further in vitro and in vivo studies. Stability of NEG8 was tested by storing it for 6 months under ambient conditions. Frequent visual inspections revealed no changes in appearance and no phase separation was noted. No significant difference was recorded for PS, PDI, or ZP (*p* < 0.01); the measured values were 29.49 ± 2.5 nm, 0.42 ± 0.06, and −19.43 ± 2.1 mV for PS, PDI, and ZP, respectively. These results indicated the stability of the optimized selected preparation: NEG8.

### 3.4. In Vitro Antibacterial Activity of NEG8

#### 3.4.1. Identification of Clinically Isolated Bacteria

##### Vitek Identification of Bacteria Isolate

The B3 isolate was identified as *Bacillus thuringiensis* using the Vitek 2 system (Table 4). Hence, B3 was a spore-forming with a unique rod shape, and it was easily identifiable as a *Bacillus* using the Vitek method. However, B1 and B2 shape are popular for many organisms and could not easily identified, so a more advanced method as molecular identification test was applied.

##### Molecular Identification of Bacterial Isolates

**Figure 4:**
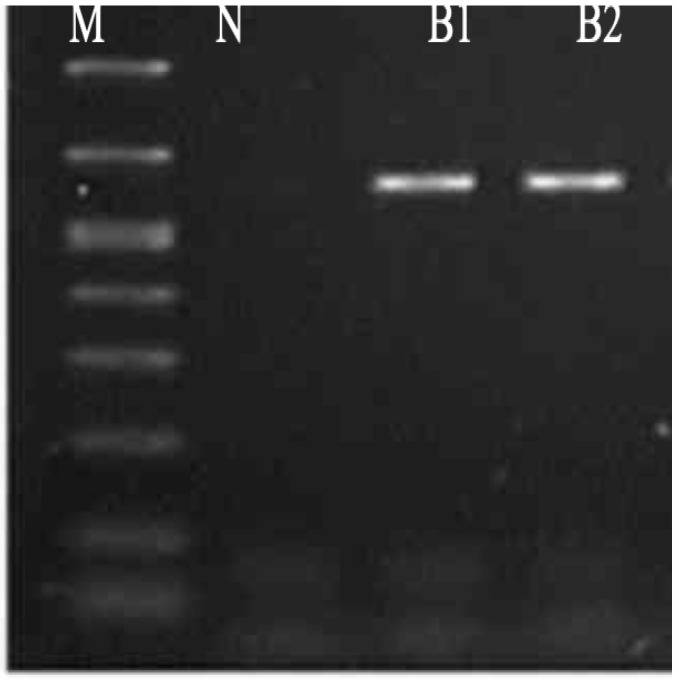
PCR product photo (PCR results using 16 s primer).

The results of electrophoresis showed no contamination or degradation of isolated samples. The first well contained the negative kb DNA ladder (M), and the second well contained the negative control to detect any contamination (N). Figure 4 demonstrates the PCR results for B1 and B2. The 16S rDNA sequence data indicated that B1 was per identical closely related (100%) to the strain *Pseudomonas stutzeri* and the B2 isolated is *Enterococcus faecium* (99.18%). These results were observed in the neighbor-joining tree patterns (Figure S1) [supplementary file].

#### 3.4.2. Sensitivity Test, MIC and MBC Assays of NEG8 against Bacterial Isolates

Our research exhibited antibacterial activities of tested NEG8 against the clinical isolates (B1, B2, and B3), but their activity was quite divers.

The following tendency of microbial sensitivity to NEG8 was observed: *Enterococcus faecium* (B2) > *Bacillus thuringiensis* (B3) > *Pseudomonas stutzeri* (B1) (Table 4). The results indicated that the the MIC of B1 and B3 was 1.25 mg/mL, while the MIC of B2 was 0.625 mg/mL. The MBC of B1 and B3 was 5 mg/mL, and it was 2.5 mg/mL for B2 (Table 5).

These results are in accordance with previous literatures that reported the inhibitory effect of clove oil against *Enterococcus* and *Pseudomonas* species [40]. Moreover, Nzeako et al. showed that thyme and clove oils possessed a remarkable antimicrobial activity against *Pseudomonas* bacterial species [41]. The high phenol content of thymol (56%) and eugenol (94%) (Table 2) can be attributed to the potent antimicrobial activity of the NEG8 [42].

#### 3.4.3. Biofilm Formation, Quantification, and Percentage of Inhibition by NEG8

**Figure 5:**
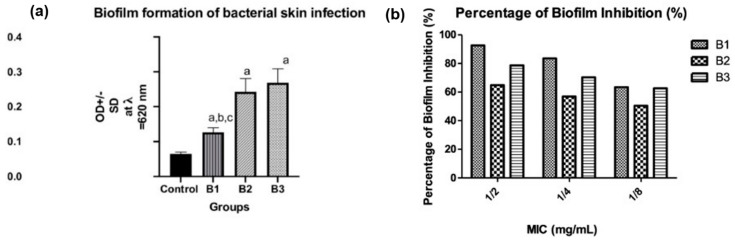
(**a**) Types of biofilm formation of isolated bacteria. Negative (OD ≤ ODc), weak (ODc ≤ OD ≤ 2ODc), moderate (2ODc < OD ≤ 4ODc), and strong biofilm production (4ODc < OD) OD = optical density (a: significant when compared to control at *p* < 0.05, b: significant when compared to B1 at (*p* < 0.05), c: significant when compared to B2 at *p* < 0.05). (**b**) The percentage of biofilm inhibition.

Microbial biofilms are constructed by bacterial communities to form complex structures that facilitate their surface adherence, permit pathogen spreading, and prevent the diffusion of antibiotic to bacterial cell wall. Therefore, it is a potential contributor to pathogen antibiotic resistance [43]. The advent of novel strategies for developing an effective biofilm inhibitor rather than conventional treatments has gained a great deal of attention. B1, B2, and B3 isolates were assessed for biofilm formation using a microtiter plate method. As per our finding, B2 and B3 exhibited strong biofilm formation, while B1 possessed a moderate biofilm formation behavior. We observed a significant difference between the control group and all isolates; moreover, the result indicated that there is a statistically measurable difference between B1 and B2 and between B1 and B3 at *p* < 0.05 (Figure 5a). The percentage of biofilm inhibition of the isolated bacteria (B1, B2, and B3) by the NEG8 was determined at 1/2, 1/4, 1/8 MICs for each isolate, and NEG8 exhibited antibiofilm activity against all isolates. B1 was inhibited by 92.70%, 83.63%, and 63.47%; B2 by 64.86%, 56.99%, and 50.46%; and B3 by 78.7%, 70.3%, and 62.6% at 1/2, 1/4, and 1/8 MICs respectively (Figure 5b). These data agreed with that of Sharma et al., 2020, who indicated that thyme and clove oil inhibited Gram-negative *E. coli* biofilm formation by 93.43% [44].

### 3.5. In Vivo Antimicrobial Potential of NEG8

#### 3.5.1. Rat Behavioral Alterations in Open Field Test

**Figure 6:**
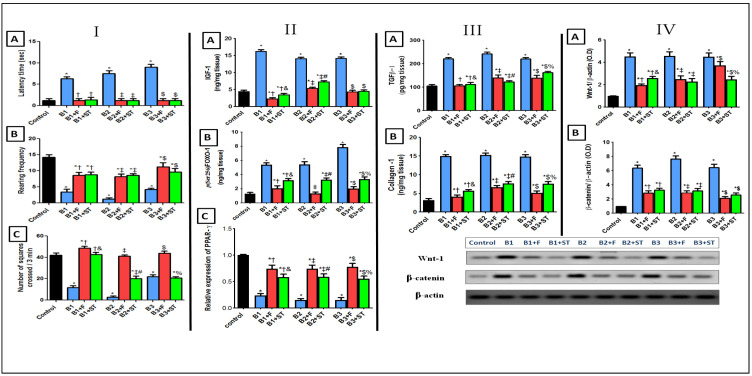
The behavioral changes in an open field test on (**A**) latency time, (**B**) rearing frequency, and (**C**) number of squares crossed/3 min (panel **I**). Contents of (**A**) IGF-1, (B) FOXO-1, and (**C**) protein expression of PPAR-γ (panel **II**). Contents of (**A**) TGFβ-1 and (**B**) collagen (panel **III**). Protein expression of (**A**) Wnt-1 and (**B**) β-catenin pathway (panel **IV**). Values are presented as mean (*n* = 10) ± SD and statistical analysis was carried out using one-way ANOVA followed by Tukey’s post hoc multiple comparison test. As compared with control (*), B1 (†), B1 + F (&), B2 (‡), B2 + F (#), B3 ($), and B3 + F (%) treated groups; *p* < 0.05. B1: *Pseudomonas stutzeri*, B2: *Enterococcus faecium*, B3: *Bacillus thuringiensis*, F: NEG8, ST: standard Adapalene gel.

The results of the open field test point to skin infection–related disarray in the spontaneous locomotor and exploratory activities, as shown in Figure 6 panel I(A). All the bacteria (B1, B2, and B3) treated groups have prolonged latency time (6.3, 7.5, and 9 s, respectively). Moreover, animals also exhibited a significant decrease in (B) rearing and (C) numbers of squares crossed/3 min when compared with the healthy control group. However, the epicutaneous application of the NEG8 for 4 days enhanced behavioral outcomes, prevented the depressive effect of bacteria by decreasing latency time, and increasing both rearing and ambulation frequencies. It is worth noting that the NEG8 has a significant altering behavioral activity compared to the ADA standard and even precedes that of control in concern with number of squares crossed/3 min that reflected the enhancement in rat behavior outcome. Moreover, to the authors’ knowledge, this study is the first experimental animal model assessing the behavior outcomes.

#### 3.5.2. IGF-1/FOXO-1 and PPAR-γ Expression

Insulin-like growth factor-1 (IGF-1) and its major downstream Forkhead box protein O-1(FOXO-1) are involved in the pathogenesis and development of skin infections [45,46]. PPAR-γ is one of the main functional isoforms of the PPAR family members and is expressed in the normal sebaceous glands that regulate human sebum production, and with a critical role in acne development [47]. As shown (Figure 6, panel II), all rats injected by different bacterial isolates acquired an immense increase in (A) IGF-1 level, then FOXO-1 nuclear activity decreased; by inducing phosphorylation at the Ser256 site and down-regulate PPAR-γ expression.

On the contrary, IGF-1 level were decreased and PPAR-γ expression was elevated after the application of NEG8. This decreased FOXO-1 phosphorylation, thereby increasing its nuclear translocation and activity. In terms of IGF-1/PPAR-γ/FOXO-1 axis, it was previously suggested that PPAR-γ may regulate FOXO-1 by modulating PI3k-AKT [48]; accordingly, NEG8 increased PPAR-γ expression and decreased IGF-1 levels, therefore hindering AKT activity and indirectly increasing FOXO activity [49].

#### 3.5.3. TGFβ-1 and Collagen

TGFβ-1 is an important pro-inflammatory cytokine [50]. Figure 6 panel (III) depicts that all the clinically isolated bacteria showed an increase in transforming growth factor β-1 (TGFβ-1) that sequentially increased collagen production [51]. However, the application of NEG8 on infected areas prevented collagen production and enhanced rat behavioral outcomes (by inhibiting their influential activator, TGFβ-1) more than the ADA standard. This effect may be attributed to the ability of the formula to decrease IGF-1 and increase PPAR-γ.

#### 3.5.4. Wnt-1/β-Catenin Expression

Wnt/ β-catenin plays a crucial role in various cellular functions [52]. All the positive control groups activated this signaling (Figure 6 panel IV), as signified by the sharp increase in the (A) Wnt-1 protein expression, which was approximately 4.5 times higher than the control group. This increase was associated with a 6.4-fold increase in (B) free β-catenin expression. Turning on this cue by all the injected bacteria may be due to elevated TGFβ-1, where previous study reported that TGF-β1 and canonical Wnt/β-catenin signaling stimulated each other via the PI3K/Akt axis [53]. The beneficial effect of NEG8 was reflected by switching off this pathway; it halted bacterial effects, decreased Wnt and subsequently β-catenin expression. This effect may have been mediated by PPAR-γ modulation, PPAR γ-agonists were shown to decrease free β-catenin levels and inhibit its nuclear translocation through activation Wnt cue inhibitors [53]. The application of NEG8 exerted higher anti-inflammatory protection by decreasing protein expression of Wnt in B1- and B3-treated groups more than the ADA standard similarly treated one.

#### 3.5.5. IL-6, IL-10, and p(Tyr (1007/1008))JAK-2/p(Tyr705)STAT-3 Signaling Pathway

**Figure 7:**
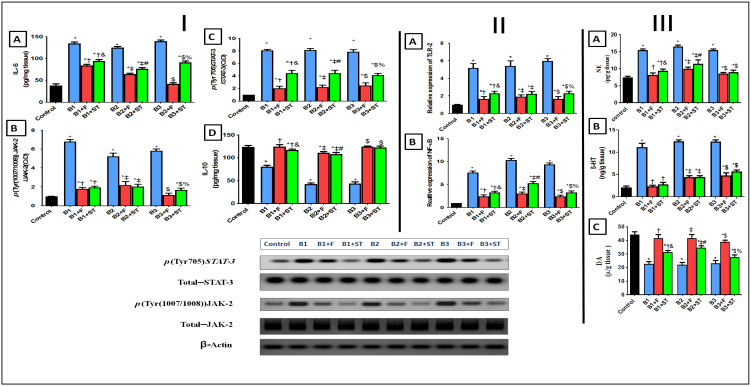
Changes in contents and protein expression of (**A**) IL-6, (**B**) *p*(Tyr (1007/1008))JAK-2 (**C**) *p*(tyr705)STAT3 and (**D**) IL-10 (panel **I**). Protein expression of (**A**) TLR-2 and (**B**) NF-κB (panel **II**). Changes in contents of (**A**) NE, (**B**) 5HT, and (**C**) DA (panel **III**). Values are presented as mean (*n* = 10) ± SD. Statistical analysis was done using one-way ANOVA followed by Tukey’s post hoc multiple comparison test. As compared with control (*), B1 (†), B1 + F (&), B2 (‡), B2 + F (#), B3 ($), and B3 + F (%) treated groups; *p* < 0.05. B1: *Pseudomonas stutzeri*, B2: *Enterococcus faecium*, B3: *Bacillus thuringiensis*, F: NEG8, ST: standard Adapalene gel.

JAK-2/STAT-3 axis is suspected to play a pivotal role in acne progression. The data in Figure 7 panel (I) illustrates the progressive stimulation of this cue that initiated by increasing of IL6 (A). The upregulation of IL6 entailed by increasing the protein expression of (B) JAK-2, downstream molecule (C) STAT-3, and decreased the content of survival cytokine (D) IL-10 (the actions intervened by all bacterial isolates). The JAK-2/STAT-3 signaling is normally triggered by cytokine receptor ligands activation (IL-6 and IL-10) [54]. In the present study, this pathway was a hazardous signaling cue, wherein in the bacteria-treated group, IL-6 was increased along with the JAK-2/STAT-3 molecules, which in turn decreased the content of the survival anti-inflammatory cytokine IL-10. However, NEG8 exerted protective measures by reverting these effects and increasing level of defending cytokine IL-10. This action pathway of NEG8 supersedes the effect of the ADA drug at different points in this trajectory.

#### 3.5.6. TLR-2 and p(Ser536) NF-κBp65 Expression

Besides these mechanisms, exaggerated inflammatory cascades are involved in the acne pathophysiology [55,56]. As shown in Figure 7 panel II, different microbial injections resulted in myriad of effects triggered by (A) TLR-2 and culminated by increasing the protein expression of parent inflammatory transcription factor (B) NF-κBp65 as well as IL-6. However, the IL-10 levels were decreased when compared with the normal control animal group. These observations agreed with data from a previous study showing that microbial agents triggered cytokine responses and NF-κB expression via toll-like receptors (TLR-2) [57].

Moreover, the enhanced transcription factor NF-κB that accounts for the formation of other proinflammatory markers can also re-activate NF-κB to cause chemokines gush along with a reduction in the protective anti-inflammatory IL-10 expression [58,59]. Post application of NEG8, the results referred to the decrease in TLR-2 and NF-κBp65 upshot in comparison with the solo bacteria treated groups. Nonetheless, NEG8 also showed higher aptitudes as anti-inflammatory more than the standard drug-treated one. Notably, four mechanistic actions putatively explained the anti-inflammatory effects of NEG8: (i) elevation of IGF-1 and pFOXO-1 levels, (ii) inhibition of NF-κB activity, (iii) stimulation of β-catenin targets genes, (iv) JAK/STAT trajectory intercedes to mediate the anti-inflammatory effect. Moreover, formerly it was implied that the activation of inflammatory mediators by TLR-2 agonist, such as IL-6, is dependent on NF-kB and JAK-STAT signaling pathways.

#### 3.5.7. Changes in Norepinephrine (NE), Serotonin (5-HT), and Dopamine (DA) Levels

In the present study, all the infected rats showed a disturbance in monoamines (Figure 7 panel III) as verified by high levels of both (A) NE and (B) 5-HT. This effect was reflected in (C) DA levels which were decreased in comparison with the control group. The direct role of these monoamines in initiating or aggravating skin infections remains unclear. It was previously shown that various types of physical stresses increase central NE [60] and 5-HT [61] levels. Furthermore, stress and depression promoted inflammatory responses that interact with many of the pathophysiological domains, particularly neuroendocrine functions, and behavior outcomes [62]. From our observations, NEG8 not only counteracted this menace but also restored normal values especially against the B1 (*P. stutzeri*) treated group. NEG8 exerted more significant effect than standard drug ADA concerning levels of DA and NE. These data suggested that restoration of near normal monoamines levels by the NEG8 was a key factor underpinning reduced inflammatory marker levels and improved behavioral sequel.

#### 3.5.8. The Effect of NEG8 on Histopathology

**Figure 8:**
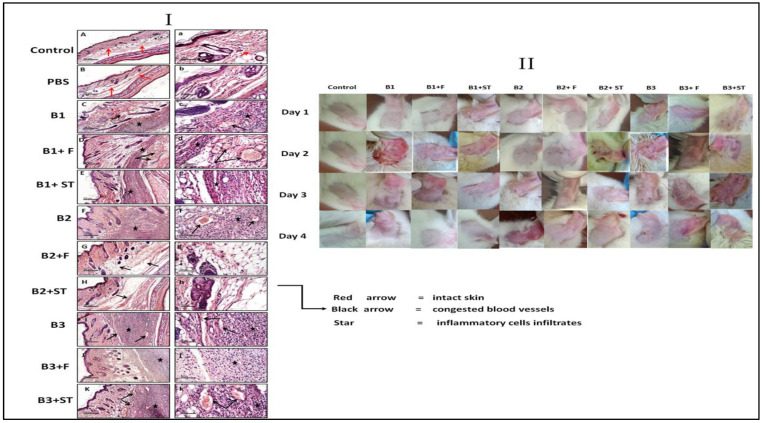
Representative images of histopathological photomicrographs alterations (panel **I**) and the skin irritation potential of NEG8 against clinically isolated bacteria (panel **II**). Panel I: compared to the (**A**,**a**) sections of normal intact skin of left ear and the (**B**,**b**) sections of right ear injected PBS, (**C**,**c**) sections showed disrupted skin morphology after B1 injection. In the same pattern, photomicrographs of B2, inoculation (**F**,**f**) and B3 inoculation (**I**,**i**) showed similar results to B1. However, sections of the left ear of animals received F for four days; (**D**,**d**; B1 + F) and (**G**,**g**; B2 + F) enhanced the skin morphology even better than standard sections of (**E**,**e**; B1 + ST) and (**H**,**h**; B2; + ADA). However, the sections treated by F (**J**,**j**; B3 + F) and the standard (**K**,**k**; B3 F + ST) did not save the skin morphology as both showed persistence of the same records as their corresponding positive control group B3 (**H**,**E**; 50 μm and 200 μm). B1: *Pseudomonas stutzeri*, B2: *Enterococcus faecium*, B3: *Bacillus thuringiensis*, F: NEG8, ST: standard Adapalene gel (ADA).

The impact of the different bacteria on histopathological of the left ear and the surrounding areas was investigated. As per our data (Figure 8, panel I) it was revealed wide diffuse areas of inflammatory cell infiltrates in subcutaneous tissue (star symbol in panel) accompanied by moderate infiltrates in the dermal layer and moderately congested blood vessels (red arrow) when compared with PBS injected ears (negative control). However, the epicutaneous application of NEG8 for 4 days displayed anti-microbial and anti-inflammatory effects against B1 (*P. stutzeri*) and B2 (*E. faecium*) groups.

This application enhanced skin appearance (panel II). In the GIIa (*P. stutzeri* + NEG8) group, a moderate reduction in inflammatory cell infiltrates was observed but with a persistence of congested blood vessels (red arrow). However, in the ADA treated group, GIIb (*P. stutzeri* +ADA) showed persistence of the same records as the GII (*Pseudomonas stutzeri: B1*) group. NEG8 exerted its protective effect in GIIIa (B2; *E. faecium* +NEG8), as evidenced by the well-organized morphological structures in different skin layers, with minimal inflammatory cell infiltrates and moderate subcutaneous edema (red arrow). Moreover, GIIIb (B2; *E. faecium* + ADA) showed significant improvement with minimal inflammatory cell infiltrates as well as few congested blood vessels (red arrow) when compared with GIII (B2; *E. faecium*) animals’ group. Nevertheless, NEG8 and even the ADA treated group did not save the skin morphology as their corresponding positive control group (B3; *B. thuringiensis*), where both showed persistence of the same records (Figure 8, panel I, II).

**Figure 9:**
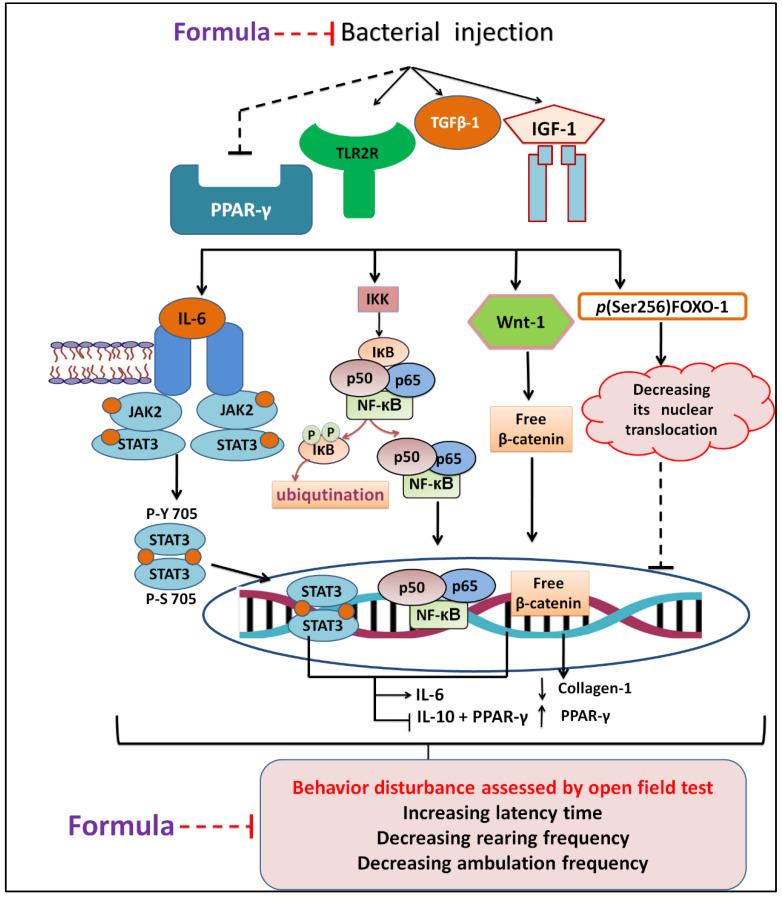
Scheme revealing the possible in vivo changes, different signaling pathways involved with skin infection, and protective effect of the NEG8 (thymol/clove).

We also observed that *P. stutzeri* increased the IGF-1 and induced higher level of skin infection more than *E. faecium* and *B. thuringiensis,* while *B. thuringiensis* triggered behavior disturbance more than *P. stutzeri* and *E. faecium* isolates. On the other side, the *E. faecium* potentiated tgfb, TLR, and NF-kB inflammatory markers more than *P. stutzeri* and *B. thuringiensis*. In contrast, *P. stutzeri* increased the JAK inflammatory pathway more than the other two bacteria. All isolated exerted the same significant effects on PPAR- γ and Wnt when compared with controls. After treatment with NEG8, no differences in latency time were observed between animal groups. Moreover, NEG8 was more effective against *P. stutzeri* than *E. faecium* and *B. thuringiensis* with respect to monoamines, TGFB-1, and Wnt parameters, indicating that NEG8 can alters inflammation progression and infection behavioral disruption state. A schematic diagram outlining the possible in vivo signaling pathway therapeutic mechanisms is shown in Figure 9. To our knowledge, this is the first report that sheds light on the effect of specific types of bacteria on biological molecular signals.

Our results are in accordance with previous studies that ascribed the use of EOs as a promising antimicrobial agent against drug resistant pathogens [63]. *Pectis substriata* EO exerted a potential activity against resistant *staphylococcus strains* [64]. The antibiotic-like action of EOs is attributed to the biofilm inhibition [65], membrane disruption and efflux suppression action mechanism [66]. Moreover, inhibition of inflammatory cytokine and suppression of superoxide production were reported as the mode of antimicrobial action of terpinen-4-ol tea tree oil [67]. Additionally, the citron EO showed a food-borne bactericidal activity through lysis of the bacterial cell wall, inhibition of microbe growth rate, and permitting the leakage of microbe’s intracellular ingredient [68].

Recently, the preparation of nanomaterials (NMs) represents an efficient strategy that implements a unique bactericidal activity that counters microbial resistance. Nitric oxide–releasing-NMs, chitosan-based-NMs, metallic-NMs, and multiple antibiotics loading-NM_S_ are different nano-forms that play a critical role in interacting with the bacterial cellular system and serve as an antibiotic alternate [69]. Currently, there are limited studies that used nano-EOs to compete bacterial resistance [70]. However, the nano-emulsion of oregano oil showed acne healing and in vitro antimicrobial activity [71]. Besides, cardamom-chitosan nano-encapsulation recorded a potent efficacy against antibiotic-resistant bacterial pathogens (β lactamase producing *Escherichia coli* and methicillin resistant *Staphylococcus aureus*) [72]. To the authors’ knowledge, most of the EOs and nano-EOs antimicrobial studies have failed to provide definite information on their mechanisms of action at the molecular level [73]. However, for the first time, our research explored the antibiotic non-antibiotic insights of nano-thyme/clove EOs (NEG8) against acne drug-resistant microbes.

In summary, NEG8 showed a bacteriostatic and biofilm inhibition activity. The application of NEG8 enhanced the rat behavioral outcomes, which were mirrored by monoamines levels. It also impacted on important signaling events induced by isolated bacteria during skin infection through modulating the expression of IGF-1, TGF-β/collagen, Wnt/β-catenin, and JAK2/STAT-3. Moreover, NEG8 up-regulated PPAR-γ receptors which perform critical role in acne/skin infection development and regulates sebum production. The anti-inflammatory activities of NEG8 were confirmed by decreasing p(Ser536) NF-κBp65, TLR-2, and IL-6 levels and increasing the threshold of protective anti-inflammatory cytokine IL-10. These results were reflected by improvements in histological structures and skin morphology. Hence, NEG8 is considered as a new non-antibiotic therapeutic agent with multiple mechanisms that can potentially slow the rate at which resistance develops.

## 4. Conclusions

The present study indicated that NEG8 (clove/thyme NEG) exhibited dual non-antibiotic and bacteriostatic antibiotic mechanisms. The non-antibiotic effects were attributed to the potential of NEG8 to inhibit nervo-motor pathogenesis and the bacterial behavioral/inflammatory pathways. Based on these findings, we propose NEG8 as a potential cosmo-natural antimicrobial agent which improves skin morphology and reduces comedonal acne. To the authors’ knowledge, no study has comprehensively outlined the effects of EOs on the measured pathways implicated in acne treatment. Further clinical research is required to explore the application of NEG8 in clinical settings. Taken together, the study revealed the possibility of developing different nano-essential oils emulgel with a multi-mechanism microbial resistance tackling strategy.

## Figures and Tables

**Table 1:** Compositions of the prepared nano-emulsion systems (each formula was loaded with 30 mg/mL of 1:1 clove/thyme mixture) and observations of visual inspection.

Surf. Ratio	Formula Code	Oil	Surfactants		Water	Visual Appearance
			Tween 80	Cremophor RH40		
(1:1)	NEG1	10	30	30	30	turbid
	NEG2	20	30	30	20	gel
	NEG3	30	30	30	10	fluid
(2:1)	NEG4	10	40	20	30	turbid
	NEG5	20	40	20	20	gel
	NEG6	30	40	20	10	fluid
(3:1)	NEG7	10	45	15	30	turbid
	NEG8	20	45	15	20	gel
	NEG9	30	45	15	10	fluid

**Table 2:** GC/MS standardization and authentication of essential oil samples.

Tested Oil	Major Area %	Rt	M.wt	Molecular Formula	Compound	Fragmentation Pattern
Garlic	40.87	12.17	178	C_6_H_10_S_3_	Allyl trisulfide	39, 41, 73, 113, 178
Eucalyptus	48.88	8.65	152	C_10_H_16_O	2-Bornanone	41, 55, 69, 81, 95, 108, 152
Lavender	39.39	7.41	154	C_10_H_18_O	L-Linalool	55, 69, 71, 80, 93, 121, 136
Cinnamon	60.97	11.73	132	C_9_H_8_O	Cinnamaldehyde	51, 77, 78, 103, 131, 132
Thyme	56.62 *	11.85	150	C_10_H_14_O	Thymol	77, 91, 115, 117, 135, 150
Lemongrass	52.46	5.86	136	C_10_H_16_	D-Limonene	41, 53, 67, 93, 107, 121, 136
Cumin	34.77	6.47	136	C_10_H_16_	Terpinene	41, 77, 79, 91, 93, 105, 121, 136
Geranium	36.27	10.3	156	C_10_H_20_O	Citronellol	41, 55, 69, 82, 95, 109, 123, 138, 156
Clove	94.13 *	13.25	164	C_10_H_12_O_2_	Eugenol	55, 77, 91, 103, 121, 131, 149, 164
Peppermint	23.90	9.26	156	C_10_H_20_O	Menthanol	41, 43, 55, 71, 81, 95, 123, 138, 156

* Refers to the phenol’s concentration (thymol and eugenol) in NEG8.

**Table 3:** Characteristics of the prepared nano-emulsion gels.

Formula Code	PS (nm)	PDI	ZP (mV)
NEG2	52.44 ± 3.22	0.413 ± 0.00	−12.40 ± 0.70
NEG5	36.43 ± 1.63 ^a^	0.260 ± 0.06 ^a^	−12.50 ± 2.26
NEG8	33.46 ± 1.31 ^a^	0.315 ± 0.05	−14.2 ± 2.31
NEG8 (6 months storage)	29.49 ± 2.51 ^a,b^	0.42 ± 0.06 ^b^	−19.43 ± 2.10 ^a,b,c^

All values are expressed as mean ((*n* = 3), ±SD); the difference is significant at *p* ≤ 0.05 from: NEG2 ^a^, NEG5 ^b^, NEG8 ^c^.

**Table 4:** Biochemical details of identified B3 (*Bacillus thuringiensi)* by Vitek2 compact system.

1	BXYL	-	3	LysA	-	4	AspA	-	5	LeuA	+	7	PheA	+	8	ProA	-
9	BGAL	-	10	PyrA	+	11	AGAL	-	12	AlaA	-	13	TyrA	-	14	BNAG	+
15	APPA	+	18	CDEX	-	19	dGAL	-	21	GLYG	-	22	INO	-	24	MdG	-
25	ELLM	-	26	MdX	-	27	AMAN	-	29	MTE	-	30	GlyA	-	31	dMAN	-
32	dMNE	-	34	dMLZ	-	36	NAG	+	37	PLE	-	39	IRHA	-	41	BGLU	+
43	BMAN	-	44	PHC	-	45	PVATE	+	46	AGLU	-	47	dTAG	-	48	dTRE	+
50	INU	-	53	dGLU	+	54	dRIB	+	56	PSCNa	-	58	NaCl	+	59	KAN	-
60	OLD	-	61	ESC	+	62	TIZ	-	63	POLYB	+	1					

**Table 5:** Sensitivity test (mm), MIC, MBC (mg/mL) of NEG8 against identified bacterial isolates.

Bacterial Name	Zone of Inhibition	MIC	MBC
*Pseudomonas stutzeri* (B1)	19	1.25	5.0
*Enterococcus faecium* (B2)	25	0.62	2.5
*Bacillus thuringiensis* (B3)	23	1.25	5.0

## Data Availability

The data presented in this study are available on request from the corresponding author.

## Supplementary Materials

The following are available online, Figure S1: Phylogenetic tree of *Pseudomonas stutzeri* (a), *Enterococcus faecium* (b).

## Sample Availability

Samples of the NEG8 are available from the authors.

## Abbreviations

5-HT: serotonin; AMR, antimicrobial resistance; B1: *Pseudomonas stutzeri*; B2: *Enterococcus faecium*; B3: *Bacillus thuringiensis*; ADA: adapalene standard; BHA, brain heart infusion agar; CFU, a colony-forming unit; DA, dopamine; DPPH, 1,1-diphenyl-2- picrylhydrazyl; EOs, essential oils, ELISA, enzyme-linked immunosorbent assay; FOXO-1, Forkhead box protein O-1; FRAP, Ferric reducing antioxidant power; GC-MS, gas chromatography–mass spectrometry; IGF-1, Insulin-like growth factor 1; IL-10, Interleukine-10; IL-6, Interleukine-6; IPM, Isopropyl myristate; JAK/STAT, Janus Kinase/Signal Transducer and Activator of Transcription; JAK2, Janus Kinase 2; MBC, Minimal bactericidal concentration; MIC, minimum inhibitory concentration; NEGs, nano-emulsion gel preparations; NEG8, nano-emulgel optimized formula; OD, Optical density; NE, norepinephrine; NF-κB, nuclear factor kappa-light-chain-enhancer; P-FOXO1(ser256), Phospho-FoxO1 (Ser256); *P. acnes*, *Propionibacterium acnes*; PBS, phosphate buffer saline; PCR, polymerase chain reaction; PDI, polydispersity index; PPARs, peroxisome proliferator-activated receptors; PPARs, peroxisome proliferator-activated receptors; p(Tyr(1007/1008)) JAK2: phosphorylated Janus activating kinase-2 at tyrosine-(1007/1008)); p(tyr705)STAT3: phosphorylated signal transducer activator factor at tyrosine 705; TLR-2: toll-like receptor-2; RIPA, radioimmunoprecipitation assay buffer; STAT3, signal transducer and activator of transcription 3; TEM, transmission electron microscope; TGF-β, transforming growth factor beta; TGF-β1, transforming growth factor beta 1; TLRs, toll-like receptors; Wnt/β-catenin pathway, the canonical Wnt pathway; ZP, zeta potential.
