# Supplementary material for: Topical Nano Clove/Thyme Gel against Genetically Identified Clinical Skin Isolates: In Vivo Targeting Behavioral Alteration and IGF-1/pFOXO-1/PPAR γ Cues
Source: Molecules. 2021 Sep 15;26(18):5608. doi: 10.3390/molecules26185608 (PMC8465895; doi:10.3390/molecules26185608)
Supplement: Supplementary file 1 [file molecules-26-05608-s001.zip › molecules-1342014-supplementary.pdf]

Supplementary material

2. Methods

2.5 Antibacterial bioassay

2.5.7 Isolation and identification of clinically isolated bacterial genes(B1&B2) using molecular techniques

2.5.7.3 Gene Sequence

Oligonucleotide primer of bacteria was supplied from metabion (Germany) : AGAGTTTGATCMTGGCTCAG, TACGGYTACCTTGTTACGACTT, target gene; 16S rRNA. PCR amplification. The concentration of extracted DNA was estimated using Nanodrop C2000 (ThermoFisher Scientific). PCR reaction using 16S primers was performed using MiniOpticon PCR device (BioRad by DreamTaq master mix PCR kit, ThermoFisher Scientific). The PCR product was purified from gel using QIAamp DNA mini kit, (Qiagen). The sequencing reaction was amplified using BigDye Terminator kit and X- terminator kit and the amplified fragments were established using 3500 Genetic Analyzer Applied Biosystems. Two directions were amplified and sequenced using 16S primers. The fragments were analyzed using FinshTV and SnapGene software followed by sequences aligned with databases on NCBI.

2.6 In-vivo anti-skin infection potential of the optimized selected NEG

2.6.4 RT-PCR analysis

The specimens from tissues were set up as described previously [33]. Quantitative converse transcriptase (qRT) PCR preliminaries were as: PPAR-γ 5'-AAGCCATCTTCACGATGCTG-3 (sense) and 5'- -TCAGAGGTCCCTGAACAGTG-3 (antisense); TLR-2, 5 GTACGCAGTGAGTGGTGCAAGT-3 (sense) and 5GGCCGCGTCATTGTTCTC-3 (antisense); and NF-KB 5'-CATTGAGGTGTATTTCACGG-3 (sense) and 5'-GGCAAGTGGCCATTGTGTTC-3(antisense); β-actin,5'GGTCGGTGTGAACGGATTTGG-3(sense)and 5′-ATGTAGGCCATGAGGTCCACC-3 (antisense). The expression levels were analyzed by Real-Time StatMiner (Integromics, Madrid, Spain). β-actin levels were used as an internal control, and fold changes were calculated by relative quantification.

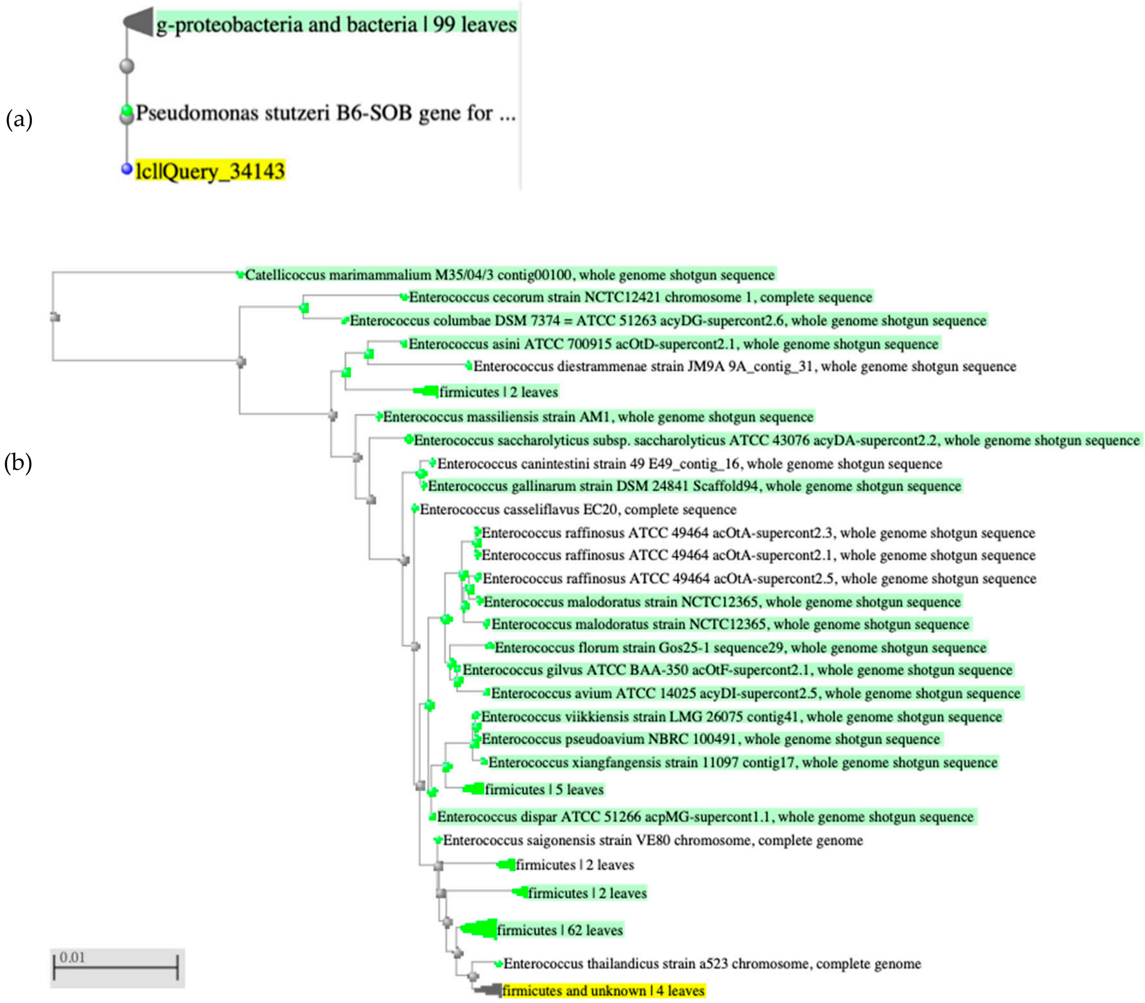

Figure S1. Phylogenetic tree of *Pseudomonas stutzeri* (a), *Enterococcus faecium (b).*
